# IgG4-related disease has a specific intestinal microbiota signature

**DOI:** 10.1016/j.ebiom.2026.106326

**Published:** 2026-06-11

**Authors:** Lisa Budzinski, Anne Elisabeth Beenken, Toni Sempert, Gi-Ung Kang, Amro Abbas, Leonie Lietz, René Maier, Mir-Farzin Mashreghi, Hyun-Dong Chang, Tobias Alexander

**Affiliations:** a German Rheumatology Research Centre, Berlin – A Leibniz Institute, Berlin, Germany; b Department for Cytometry, Institute of Biotechnology, Technische Universität Berlin, Berlin, Germany; c Department of Rheumatology and Clinical Immunology, Charité - Universitätsmedizin Berlin, Berlin, Germany

**Keywords:** IgG4-related disease, Intestinal microbiome, Microbiota, Flow cytometry

## Abstract

**Background:**

While the intestinal microbiome has been implicated in Immunoglobulin-4 related disease (IgG4-RD), it remains poorly characterised. Therefore, we performed a comprehensive microbiome characterisation to identify disease-specific alterations.

**Methods:**

In this cross-sectional study, cryopreserved stool samples from 28 patients with IgG4-RD were characterised by 16S rRNA gene sequencing and by multiparameter microbiota flow-cytometry to determine their taxonomic composition and phenotype at the single cell level. These data were evaluated in comparison with 24 healthy controls (HC) and assessed for their potential to classify IgG4-RD using random forest classification, with an independent validation cohort (12 IgG4-RD, 12 HC).

**Findings:**

Patients with IgG4-RD exhibited reduced taxonomic diversity and disease-specific alterations in the microbiome compared to HC, characterised by significantly elevated levels of several species within the Bacillota phylum. These taxonomic alterations classified patients and HC with an AUROC of 0.87 (95% CI: 0.77–0.97) but showed reduced performance in the validation cohort (AUROC 0.58, 95% CI: 0.29–0.87). Flow cytometry revealed distinct phenotypic microbiota alterations, robustly distinguishing patients with IgG4-RD from HC in both the training (AUROC 0.9, 95% CI: 0.81–0.99) and validation cohort (AUROC 0.78, 95% CI: 0.59–0.97). The IgG4-RD microbiota were predominantly DNA-low and showed no enhanced endogenous IgG4 coating, neither natively nor after *in vitro* incubation with autologous serum.

**Interpretation:**

Our study revealed specific alterations in the intestinal microbiota on taxonomic and phenotypic level in IgG4-RD, which potentially reflect different mechanisms of adaptations of the gut microbiota to immune disturbances specific to IgG4-RD. We provide proof-of-concept that this “microbiota fingerprint” may be suitable to identify IgG4-RD in a machine-learning approach and may provide important insights into the complexity of intestinal microbiota alterations in IgG4-RD.

**Funding:**

This work was supported by grants from Rolf M. Schwiete Foundation, DFG (German Research Foundation), Innovative Medicines Initiative 2 Joint Undertaking (3 TR), and EFRE-Project.

**Research in context**

**Evidence before this study**

IgG4-RD has been linked to environmental factors and initial data indicated alterations in the intestinal microbiome. Existing data on the intestinal microbiome in IgG4-RD are sparse, mostly include biliary/pancreatic disease and are limited to 16S rRNA sequencing.

**Added value of this study**

By combining conventional 16 S rRNA sequencing with single cell-based flow cytometry and machine learning, we found microbiome alterations in IgG4-RD. Taxonomic and phenotypic changes in the intestinal microbiome were independent from the clinical phenotype of patients with IgG4-RD. We provide proof-of concept that a “microbiota fingerprint” could be suitable to classify IgG4-RD in a machine-learning approach.

**Implications of all the available evidence**

Our study provides important insights into the complexity of intestinal microbiota alterations in IgG4-RD, which comprise changes in the composition of the intestinal microbiota but also their phenotypic properties, potentially reflecting different mechanisms of adaptations of the gut microbiota to immune disturbances specific to IgG4-RD. Faecal sample analysis may be suitable to identify patients with IgG4-RD, complementing standard diagnostic tools in the future.

## Introduction

IgG4-related disease (IgG4-RD) is a rare, chronic condition characterised by tissue infiltration of lymphocytes and IgG4-expressing plasma cells with specific features of fibrosis.^1^ The disease is usually indolent and slowly progressive with a highly heterogeneous clinical phenotype, which often poses a diagnostic challenge for clinicians. In fact, the majority of patients have already acquired permanent organ damage at the time of the diagnosis.^2^ Most commonly involved organs include the pancreas, salivary glands, the retroperitoneum, aorta and the bile tract, but nearly any organ system or anatomic site can be affected.^3^ While the aetiology of the disease is not fully understood, recent data have suggested a prominent role of environmental triggers, i.e. higher disease prevalence among blue-collar workers^4^ and smoking history^5^ among affected patients.

Many studies have indicated that interactions of the intestinal microbiota with the hosts’ immune system may contribute to the development and perpetuation of autoimmune^6^^,^^7^ or metabolic^8^ diseases. In IgG4-RD, microbiota analyses are limited and have focused primarily on autoimmune pancreatitis.^9^^,^^10^ One study recently investigated the gut microbiota in all phenotypes of IgG4-RD, using metagenomic sequencing of stool samples from patients with IgG4-RD and systemic sclerosis. They found an abundance of opportunistic pathogenic *Clostridium* as well as oral *Streptococcus* species combined with a decrease of *Alistipes, Bacteroides* and butyrate-producing species along with a reduced alpha-diversity.^11^ Similarly, an altered faecal microbiome and metabolome was identified in IgG4-related sclerosing cholangitis with increased levels of *Streptococcus* and reduced *Blautia* and *Lachnospiraceae,* suggesting unique metabolic host-microbe interplays that may be involved in disease pathogenesis.^12^ However, to the best of our knowledge, no study has directly addressed the interplay between the immune system and the microbiome in IgG4-RD.

To complement traditional high-throughput microbiome profiling by 16S rRNA sequencing, we recently described the microbiome analysis by single-cell microbiota phenotyping with multiparametric flow cytometry.^7^^,^^13^, ^14^, ^15^, ^16^ With this technique, we can assess the microbial community structure and diversity with quantitative DNA staining and light scatter measurement. We quantitatively and isotype-specifically analyse the coating of bacterial cells with host immunoglobulins representing their immune recognition and the intestinal immune response in general. In addition, we determine the expression of specific surface sugars, namely galactose, mannose and N-acetyl-glucosamine, by bacteria with sugar-specific plant-derived lectins. We have previously shown that lectin-bound fractions can reflect intestinal microbiota changes not represented on taxonomic level,^7^ and that the microbiota phenotyping approach may reveal adaptation processes of the bacteria to their complex microenvironment in the intestinal tract specific to a disease, which holds the potential to reveal mechanisms of intestinal bacteria contributing to disease. We here compared intestinal microbiota samples of patients with IgG4-RD and healthy controls to identify significant differences in the phenotypic microbiota fingerprints or the taxonomic microbiome composition. The results were refined by machine-learning, comparable to biomarker identification, and we demonstrate their potential for IgG4-RD classification by random forest modelling, which we propose as a compelling application to support the challenging diagnosis of IgG4-RD.

## Methods

### Ethics

All participants gave written informed consent prior to sample collection according to the approval of the local ethics committee of the Charité—Universitätsmedizin Berlin, Germany (EA4/014/20 and EA2/113/20).

### Subjects

In this cross-sectional study, we recruited 28 patients with IgG4-RD from the Department of Rheumatology and Clinical Immunology at the Charité—Universitätsmedizin Berlin who met the 2019 ACR/EULAR classification criteria for IgG4-RD^3^ between December 2020 and October 2021 in a training cohort. All experiments were repeated with an independent validation cohort of 12 patients, who were recruited between December 2020 and December 2023. Sex-matched healthy controls were recruited during the same period, excluding those with ongoing infections, known autoimmune diseases or intake of systemic immunosuppressive medication. Sex was self-reported by participants.

### Sample collection and processing

All participants provided a stool sample of about 1–2 g, collected in a stool sampling tube (Sterilin™, Thermo Fisher Scientific). The samples were immediately transferred to 4 °C and kept at that temperature for a maximum of 96 h before further processing. The time between stool collection by the participants and transfer to 4 °C ranged between 4 and 24 h. Stool samples were processed as previously described.^7^^,^^16^ In short, each sample was diluted in autoclaved and 0.2 μm sterile-filtered PBS (Steritop® Millipore Express®PLUS 0.22 μm, Cat. No: 2GPT05RE) to a concentration of 100 mg/ml. The suspension was sequentially filtered through 70 μm (Falcon, Cat. No. 352350) and 30 μm filters (CellTrics®, Sysmex). 10 μl of each sample was subsequently stored at −20 °C for later 16S rRNA gene sequencing. The remaining sample was frozen at −80 °C in 40% glycerol with a defined OD of 0.4. For each patient and HC, 1.5 ml of serum was obtained and stored at −20 °C.

### Multiparameter microbiota flow cytometry (mMFC)

Frozen microbiota stocks of 0.4 OD were topped up with 1 ml of autoclaved and sterile-filtered PBS and centrifuged at 13,000 × *g* for 10 min, 4 °C.^7^^,^^16^ The pellet was incubated in 500 μl blocking solution containing 20 μg/ml mIgG1 (clone: IS5-21F5, Miltenyi Biotech Cat. No.: 130-106-545) and 10 μg/ml mIgG2a (clone: S43.10, Miltenyi Biotech Cat. No.: 130-106-546) in PBS for 5 min at RT. The suspension was topped with 1.5 ml PBS and subjected to another centrifugation step (13,000 × *g*, 10 min, 4 °C). The pellets were re-suspended in PBS containing 0.2% BSA (v/w) and 25 μg/m l DNase (Sigma Aldrich Cat. No. 10104159001), which was also used as staining buffer. Cell density was adjusted to 0.02–0.04 OD_690_/ml. 100 μl of the cell suspension was used per staining panel, i.e. stained with the immunoglobulin or the lectin panel. For the immunoglobulin panel, the antibodies used were anti-human IgM-Brilliant Violet 650 (clone: MHM-88, Biolegend® Cat. No. 314526), anti-human IgG-PE/Dazzle™ 594 (clone: HP6017, Biolegend® Cat. No. 409324), anti-human IgA1-Alexa Fluor 647 (clone: B3506B4, Southern Biotech Cat. No. 9130-31), anti-human IgA2-Alexa Fluor 488 (clone: A9604D2, Southern Biotech Cat. No. 9140-30). The lectins used for staining were 0.5 μg/test of Peanut Agglutinin-CF®488 (PNA, Biotium Cat. No.29060), 0.5 μg/test of Concanavalin A-CF®680 (Con A, Biotium Cat. No. 29020-1), 0.25 μg/test of Wheat Germ Agglutinin-CF®555 (WGA, Biotium Cat. No.29076-1) and 0.5 μg/test of biotinylated Solanum Tuberosum Agglutinin (STL, Biozol Cat/Biozol Cat. No. B-1165) shortly pre-incubated with 2 μl (1:50, v/v) anti-Biotin-PerCP antibody (clone: Bio3-18E7, Miltenyi Biotech Cat. No. 130-133-293). The tests were incubated for 30 min at 4 °C and subsequently topped up with 1 ml of 5 μM Hoechst solution (Hoechst33342, Thermo Fisher Scientific Cat. No. 62249) and incubated for another 30 min at 4 °C. The samples were washed with 900 μl PBS/BSA and re-suspended in fresh PBS/BSA after centrifugation for acquisition. All samples were acquired on a BD Influx*™* cell sorter (Becton–Dickinson). For each sample, 3 × 10^5^ Hoechst33342-positive events (mean fluorescence intensity >10) were recorded. We controlled the staining procedure by including a standardised microbiota sample (anchor sample) comprising a pool of different donors.

### Data processing in R

The data were processed and evaluated similarly as previously stated in detail.^7^ The flow cytometric data was clustered by SOM^17^^,^^18^ to 2025 clusters per panel and subjected to statistical comparisons as well as machine learning (RFE)^19^, ^20^, ^21^ to identify clusters that are relevant to describe the comparison of patients with IgG4-RD and controls at best. In short, normalised mMFC cluster counts and relative abundances of bacterial genera from 16 S rRNA amplicon sequencing were filtered by Wilcoxon rank-sum test, p > 0.05 (vegan package)^19^ to excluded features not contributing to the discrimination between IgG4-RD and healthy samples. RFE was performed with 10-fold cross-validation to remove features not contributing to classification with the rfeControl () function in caret package.^20^ The resulting features from the mMFC or 16S rRNA gene amplicon sequencing were used to train the respective random-forest model with 10 times repeated 10-fold cross-validation with function of train (ntree = 1500) and trainControl ()using default settings. The validation samples were used to evaluate the models' classification performance in one modelling attempt. The model performance was evaluated with the evalm () function from the MLeval package. F-score (F1)—the harmonic mean of precision and recall, summarising a classifier's ability to balance correct positive predictions against false positives and ranges between 0 and 1. A higher F1 (>0.7) indicates a better trade-off between sensitivity and positive-predictive value.

### 16S rRNA sequencing (Illumina MiSeq platform)

For 16S rRNA gene sequencing, we amplified the V3/V4 region of the 16S rRNA gene (for: TCGTCGGCAGCGTCAGATGTGTATAAGAGACAGCCTACGGGnGGCWGCAG, rev: GTCTCGTGGGCTCGGAGATGTGTATAAGAGACAGGACTACHVGGGTATCTAATCC^22^; TIB MOLBIOL Syntheselabor GmbH) directly from a microbiome sample with a prolonged initial heating step of 5 min. After the amplicon PCR the genomic DNA was removed by AmPure XP Beads (Beckman Coulter Life Science Cat. No. A63881) with a 1:1.25 ratio of sample to beads (v/v). The amplicons were checked for their size and purity on a 1.5% agarose gel, and if suitable, subjected to the index PCR using the Nextera XT Index Kit v2 Set C (Illumina, FC-131-2003). After Index-PCR, the samples were cleaned again with AmPure XP Beads (Beckman Coulter Life Science Cat. No. A63881) in a 1:0.8 ratio of sample to beads (v/v). Samples were analysed by capillary gel electrophoresis (Agilent Fragment Analyser 5200) for correct size and purity with the NGS standard sensitivity fragment analysis kit (Agilent Cat. No. DF-473). Of all suitable samples a pool of 2 nM was generated and loaded to the Illumina MiSeq 2500 system.

### Sequence alignment of Illumina MiSeq data

Paired-end reads generated by Illumina MiSeq 16S rDNA sequencing were filtered and trimmed using Trimmomactic (Version 0.39).^23^ 7 leading bases with qualities below 35 were trimmed and reads shorter than 180 bases were filtered out. Using the DADA2 (Version 1.22.0) software package,^24^ forward and reverse reads were truncated at 260 and 210 bases respectively and filtered with a minimum quality score of 12 and a maximum of 0 ambiguous nucleotides. Amplicon sequence variants (ASVs) were identified using the default settings of the DADA2 algorithm and ASVs were classified using the Silva 138.1 prokaryotic SSU taxonomic training data formatted for DADA2.^25^ After alignment of the sequences with DECIPHER (Version 2.24.0),^26^ a phylogenetic tree was computed using FastTree (Version 2.1.11).^27^ For analysis the ASVs were matched with the respective phylogenetic information, the data was processed to genus level and normalised prior to any further calculations.

### PICRUSt prediction analysis

The PICRUSt2 (Phylogenetic Investigation of Communities by Reconstruction of Unobserved States) pipeline (v2.6.0)^28^ was applied to assess the metabolic functional potential of the sampled bacterial communities. Using the amplicon sequence variant (ASV) counts and their corresponding representative sequences as input, the pipeline predicted gene family abundances and reconstructed pathway-level functional profiles based on the Kyoto Encyclopaedia of Genes and Genomes (KEGG) database. Downstream analysis of the PICRUSt-predicted pathway abundances was performed in R (v4.4.2) with the help of the phyloseq (v1.52.0)^29^ and microbiomeMarker (v1.13.2)^30^ R packages. The linear discriminant analysis effect size (LEfSe) method^31^ was used to identify differentially abundant metabolic pathways between cohorts. After normalisation of the pathway abundances to counts per million (CPM), group-wise differences were assessed using the Kruskal–Wallis test and resulting p-values were adjusted for multiple testing using the Benjamini-Hochberg false discovery rate (FDR) correction, with an adjusted significance threshold of 0.05. Linear discriminant analysis (LDA) was subsequently applied to estimate effect sizes and only features with an absolute LDA score ≥2 were retained as significantly enriched. The bar charts were generated using ggplot2 (v3.5.1).^32^

### Statistical data analysis

Statistical analyses were implemented through R (v. 4.0.3 or later versions), unless stated otherwise (Supplementary Methods). Computation of β-diversity (Bray–Curtis dissimilarity) was computed using vegdist (data, method = ”bray”) function from vegan package.^19^ The Bray–Curtis dissimilarity is a statistical metric to quantify the difference in composition between two cohorts. In our case, it is the abundance of cells in each cluster or the presence or absence of bacterial taxa and their abundance. The Bray–Curtis dissimilarity is defined by a number between 0 and 1, 0 indicating that two samples/cohorts are completely similar and 1 indicating that two samples/cohorts do not share anything. The PCoA was computed by the R base function cmdscale () on the respective distance matrix followed by Adonis test adonis () from vegan package to evaluate the variance within groups. Graphical representation of the dissimilarity of all samples by Principal Coordinates Analysis (PCoA) was plotted using ggplot2 package. Correlation analysis data were evaluated by Spearman's ρ, e. g. in stat_cor (method = “spearman”) and the statistical comparisons of data points by stat_compare_means (method = “wilcox.test”, paired = “FALSE”) using ggpubr package.

### Role of funders

The funders had no role in study design, data collection, data analyses, interpretation, or writing of the report.

## Results

### 16S rRNA gene sequencing reveals taxonomic microbiome alterations in IgG4-RD

We first characterised the taxonomic composition of the intestinal microbiome from stool samples of 28 patients with IgG4-RD and 24 HCs of the training cohort by 16S rRNA gene amplicon sequencing. The demographics and clinical characteristics are provided in Table 1.

**Table 1:** Demographic and clinical characteristics.

	Training cohort		Validation cohort		p-value^d^
	IgG4-RD (n = 28)	HC (n = 24)	IgG4-RD (n = 12)	HC (n = 12)	
Demographic variables					
Age (median years, range)	56 (29–83)	47 (26–75)	70 (47–83)	50 (26–65)	<0.01^a^
Male (n, %)	18 (64.3)	16 (66.7)	5 (41.7)	5 (41.7)	0.185^b^, ^e^
Female (n, %)	10 (35.7)	8 (33.3)	7 (58.3)	7 (58.3)	0.185^b^, ^e^
Clinical phenotype (n, %)					0.379^c^^,^^e^
Pancreato-hepato-biliary	4 (14.3)		0 (0)		
Retroperitoneal/Aortitis	6 (21.4)		1 (8.3)		
Head/Neck	10 (35.7)		7 (58.3)		
Mikulicz/systemic	8 (28.6)		4 (33.3)		
Disease variables					
Disease duration (years, range)	2.57 (0.1–15.1)		1.83 (0.1–20)		0.701^a^
IgG4 Responder Index	4 (0–14)		4 (1–12)		0.941^a^
Number of involved organs	2 (1–4)		1.5 (1–5)		0.766^a^
Number of damaged organs	1 (0–3)		1 (0–3)		0.819^a^
Treatment (n, %)^f^					
Naïve	2 (7.1)		3 (25.0)		0.149^c^
Glucocorticoids	21 (75)		7 (58.3)		0.292^b^
Prednisolone dosage in mg/d	5 (1–15)		5 (2–20)		0.214^a^
Azathioprine	3 (10.7)		3 (25)		0.341^c^
Cyclophosphamide	2 (7.1)		0		1^c^
Methotrexate	5 (17.9)		0		0.293^c^
Rituximab past 18 months	10 (35.7)		7 (58.3)		0.285^c^
Serology (n, %)					
Elevated IgG4	6 (21.4)		6 (50)		0.13^c^
Elevated IgG2	4 (14.3)		3 (25)		0.41^c^
Elevated sIL2-receptor	6 (21.4)		3 (25)		1^c^
Elevated C-reactive protein	13 (46.4)		3 (25)		0.297^c^
Low complement for C3 or C4	0		0		N/A
Elevated eosinophils	1 (3.6)		1 (8.3)		0.776^c^

Statistical analysis of patient data (training cohort *vs* validation cohort) was performed using.

N/A: not applicable/not available. Statistical comparisons between patients with IgG-RD and HCs can be found in the Table S1.

a Mann-Whitney-U Test.

b Pearson Chi–Square Test.

c Fisher's exact test.

d p-values for statistical comparisons between the IgG4-RD training cohort and the IgG4-RD validation cohort.

e Distribution of sexes and distribution of clinical phenotypes was calculated using group-wise comparisons.

f Multiple medications per patient allowed.

**Fig. 1:**
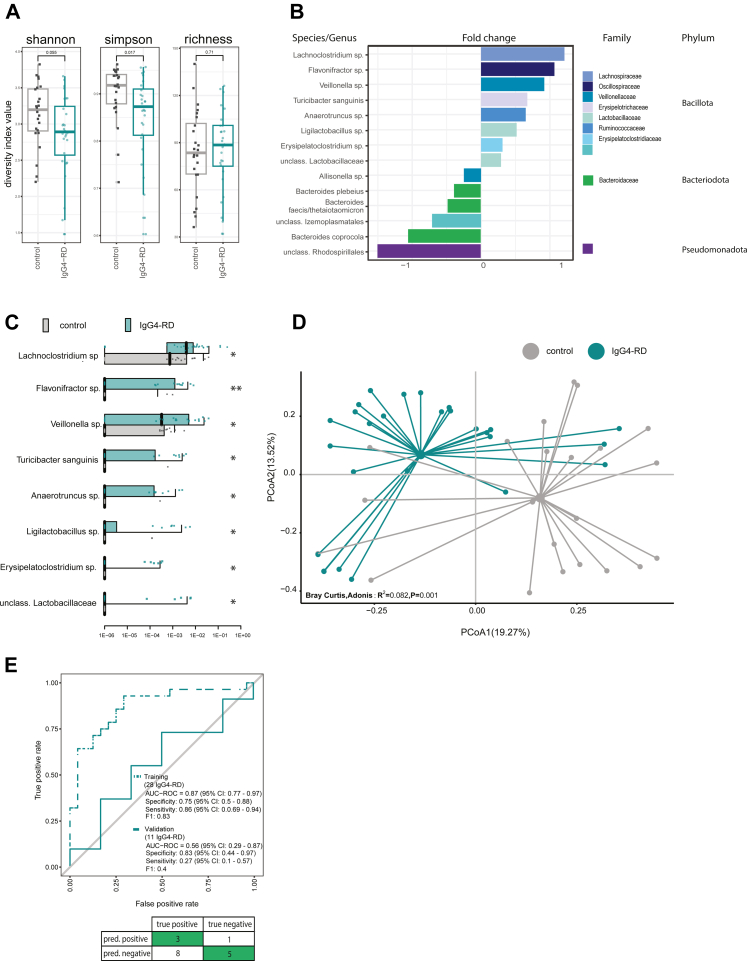
**The composition of the microbiome in patients with IgG4-RD (turquoise) is altered and reduced in diversity compared to healthy controls (grey)**. **A** Alpha diversity indices for the entire microbiome profiles of patients with IgG4-RD and healthy controls. **B** Representation of the 14 significantly different microbial taxa for the comparison IgG4-RD *vs.* healthy controls. **C** Abundance of the microbial taxa enriched in patients with IgG4-RD in the individual samples. **D** Beta-diversity represented by the dissimilarity (distance) of IgG4-RD and healthy control microbiomes according to the selected 14 microbial taxa. **E** AUROC of the random forest modelling training stage (dotted line) and for the classification of 11 patients with IgG4-RD and 6 healthy controls (solid line). Panel A–E: IgG4-RD n = 24, healthy controls n = 28; panel A, C: Mann–Whitney U test (p-values (∗ ≤ 0.05, ∗∗ ≤ 0.01, ∗∗∗ ≤ 0.001)) panel D: PERMANOVA; panel E: random forest classifier.

The IgG4-RD microbiomes displayed a decreased alpha diversity compared to healthy donors according to the Shannon (p = 0.055) and Simpson (p = 0.017) index (Fig. 1A). The richness was not significantly different between the two groups (p = 0.71), indicating that the two cohorts do not differ with regards to the number of different bacterial taxa but rather show different abundances for the same taxa. To compare the overall microbiome composition, we determined the beta diversity between the two cohorts calculated as the Bray–Curtis dissimilarity index, which is close to zero for similar microbiomes and close to 1 for distinct microbiomes. We projected this dissimilarity of samples as distances in a Principal Coordinates Analysis (PCoA) plot to present all donors and their relation to each other (Fig. S1). Specifically, taxa of the phylum Bacillota were elevated in patients with IgG4-RD, while primarily taxa belonging to the phylum Bacteriodota were decreased (Fig. 1B). *Lachnoclostridium, Flavonifractor* and *Veillonella* were genera most significantly enriched in IgG4-RD microbiomes. None of the taxa were present in all donors and their abundances differed broadly (Fig. 1C). Accordingly, the projection of the beta-diversity restricted to the selected taxa for all samples in a principal coordinates analysis plot showed a relatively large distance between individuals within each cohort, but with a significant separation of patients and controls (Fig. 1D, p = 0.001). When using this selection of taxa to train a random forest classifier model, we achieved an AUROC of 0.87 (95% CI: 0.77–0.97, specificity: 0.75 (95% CI: 0.55–0.88), sensitivity: 0.86 (95% CI: 0.69–0.94) and F1 score of 0.83, Fig. 1E). However, the classifier model could not be validated in an independently collected cohort of 11 patients with IgG4-RD (clinical characteristics provided in Table 1, one validation sample could not be amplified for 16S rRNA sequencing), performing with an AUROC 0.58 (95% CI: 0.29–0.87, specificity: 0.27 (95% CI: 0.1–0.57), sensitivity: 0.83 (95% CI: 0.44–0.97) and F1: 0.4) and identifying only 3 out of 11 patients and 5 out of 6 controls of the validation cohort correctly.

Overall, these data show that although statistically significant taxonomic differences in the microbiomes of with IgG4-RD could be identified, these were not sufficient to distinguish patients with IgG4-RD from healthy controls. In order to achieve more functional insight into the IgG4-RD microbiome, we inferred metabolic pathways using PICRUSt2 (phylogenetic investigation of communities by reconstruction of unobserved states 2)^28^ (Fig. S2). Several metabolic pathways were predicted to be differentially enriched when comparing IgG4-RD microbiomes to that of healthy controls, highlighting that changes in the functional repertoire are important to fully understand the microbiome in IgG4-RD.

### The microbiota of patients with IgG4-RD has a distinct phenotype compared to healthy controls

**Fig. 2:**
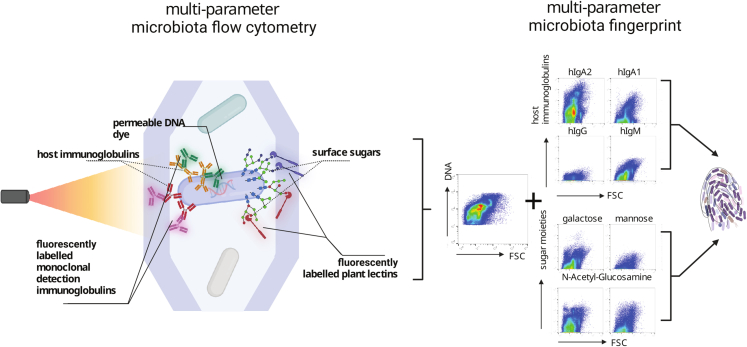
**Schematic of the single cell microbiota phenotyping approach**. Bacterial cells are isolated from stool samples and fluorescently labelled for their DNA content, host immunoglobulin coating (panel 1) and the surface expression of the indicated sugar moieties (panel 2). The labelled cells are individually analysed by flow cytometry. The obtained staining patterns are computed in a microbiota fingerprint that represents the distribution of all cell events in 11-parameters.

**Fig. 3:**
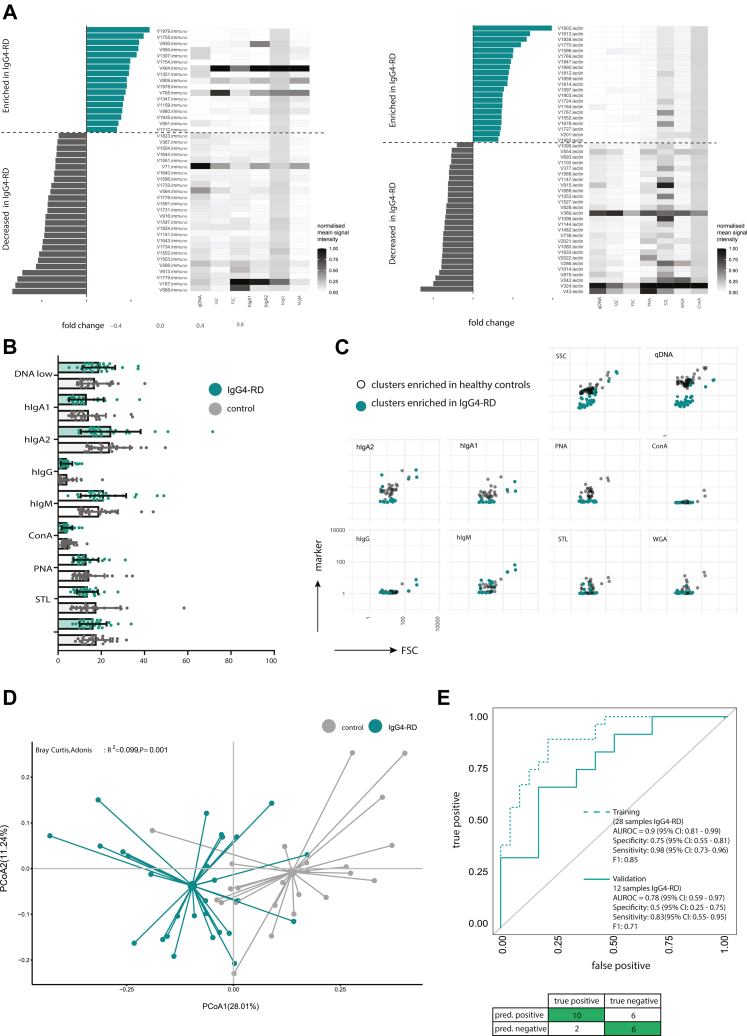
**Phenotypic features of intestinal bacteria are distinct in IgG4-RD**. Patients in turquoise, controls in grey. **A** Representation of 91 significantly different phenotypic clusters of the immunoglobulin and the lectin panel, respectively. **B** Abundance of bacteria positively stained for the indicated marker per sample according to manual gating. **C** Representation of the selected phenotypic clusters for their location in 2D plots for all markers *vs.* forward scatter signal (FSC). **D** Beta diversity of IgG4-RD and healthy control microbiota phenotypes according to the 91 significantly different clusters. **E** AUROC of the random forest model at training stage (dotted line) and for the classification of 12 patients with IgG4-RD and 12 healthy controls. Panel B: IgG4-RD n = 24, healthy controls n = 28; panel B: Mann–Whitney U test, panel D: PERMANOVA.

We next investigated the intestinal microbiota of patients with IgG4-RD on a functional and cellular level by staining the bacterial cells with surface binding reagents in two panels and measuring single cells by flow cytometry (Fig. 2). One panel was used to determine the surface coating with endogenous immunoglobulins (IgA1, IgA2, IgG and IgM) while the other assessed the expression of specific sugar moieties on the surface of the cells (mannose, N-Acetyl-glucosamine and galactose) with lectins. For the analysis of the multi-dimensional single cell data, we performed a clustering algorithm on the complete dataset, which binned the cells into 2025 clusters per staining panel, i.e. 4050 clusters in total comprising the microbial fingerprint.^7^^,^^33^ Following statistical filtering, we could identify 91 clusters, which were significantly differentially abundant between IgG4-RD *vs.* HC, comprising 43 clusters from the immunoglobulin panel and 48 from to the lectin panel (Fig. 3A). Of those, 17 immunoglobulin clusters and 21 lectin clusters were elevated in patients with IgG4-RD.

The majority of clusters enriched in IgG4-RD are composed of cells with a low signal intensity value for DNA staining (Fig. 3A and B). This is also evident when the relevant clusters different in IgG4-RD and HC are projected relative to each other according to their mean forward scatter signal (FSC) on the x-axis and the respective marker on the y-axis (Fig. 3C). The majority of selected clusters enriched in IgG4-RD were located in the low intensity regions for the DNA dye and were low in sideward scatter signal (SSC). Similarly, most of the IgG4-RD enriched clusters had low signal intensities for most of the stained markers both for immunoglobulins and lectins. However, we could identify three clusters of bacteria which exhibited increased immunoglobulin coating and forward scatter signal. HC showed primarily IgA1, IgA2 and IgM stained clusters in the FSC^low^ populations. With respect to surface glycans, IgG4-RD was characterised by lack of lectin staining, except for a several FSC^low^ clusters with prominent staining with STL indicating N-acetylglucosamine expression. HC several clusters stained by PNA and STL. Manual gating for each parameter separately did not capture the differences between IgG4-RD and HC, it did, however, reproduce the increase of DNA low events in IgG4-RD samples (Fig. 3B, Fig. S3). The frequency of PNA, STL and WGA staining showed a positive correlation with IgA1, IgA2 and IgM coating in HC (Fig. S4). In IgG4-RD, we did not observe any positive or negative correlation. Calculating the beta-diversity using the selected 91 clusters significantly improved cohort separation in the principal coordinates analysis projection of the BC dissimilarity of the samples (Fig. 3D, R^2^ = 0.099, p = 0.001) compared to the comparison using all clusters (Fig. S1B). Similar to the 16 S rRNA-based microbiome profiling, we trained a random forest model based on the selected clusters that performed with an AUROC of 0.9 (95% CI: 0.81–0.99, specificity: 0.75 (95% CI: 0.55–0.81), sensitivity: 0.89 (95% CI: 0.73–0.96) and F1: 0.85) and classified 10 out of 12 patients and 6 out of 12 controls of the validation cohort correctly with an AUROC of 0.78 (95% CI: 0.59–0.97, specificity: 0.5 (95% CI: 0.25–0.75), sensitivity: 0.83 (95% CI: 0.55–0.95) and F1: 0.71, Fig. 3E).

To assess whether immunosuppressive therapy influenced the microbial signatures, we evaluated Bray–Curtis distances between patients who had and had not received the B-cell depleting biologic therapy with rituximab using the signature classifying patients with IgG4-RD from healthy donors (Fig. S5A). Rituximab treatment was associated with separation along PCoA2, explaining 11.35% of the variance, whereas PCoA1—capturing the primary variance distinguishing IgG4RD from HC—was not affected. We additionally examined clinical phenotype (Fig. S5B), antibiotic usage within the 3 months preceding analysis (Fig. S5C), and age (Fig. S5D), as potential confounders of the IgG4-RD signature. However, none of the potential confounders impacted the IgG4-RD specific signature. Thus, the IgG4-RD microbiota is characterised by phenotypic alterations on the single cell level, which differ significantly and specifically from the microbiota of healthy controls.

### Endogenous IgG4 coating on gut microbiota is not increased in IgG4-RD

**Fig. 4:**
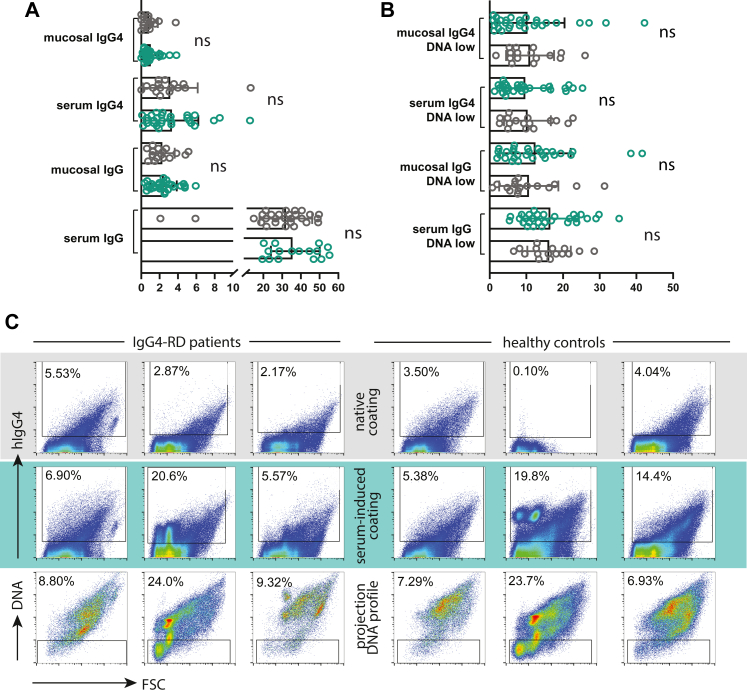
**Binding of mucosal and serum-derived endogenous IgG4 and IgG to bacteria does not differ between IgG4-RD (turquoise) and control (grey) microbiomes**. **A** Frequencies of IgG4^+^ or IgG^+^ bacteria either natively coated or after the bacteria were incubated with autologous serum. **B** Frequency of bacteria positive for IgG4 or IgG that are located in the DNA low region of the DNA profile (MFI <10). **C** Exemplary plots of 3 IgG4-RD samples and 3 healthy control samples showing the staining of bacteria for native IgG4 coating or after incubation with serum and their DNA *vs*. FSC properties. Panel A–B: IgG4-RD n = 24, healthy controls n = 28; Mann–Whitney U test.

As patients with IgG4-RD typically exhibit elevated IgG4 serum levels, we next tested for the presence of endogenous IgG4 on the bacterial surface and whether serum IgG4 of patients with IgG4-RD showed increased reactivity to the microbiota (Fig. 4). We did not find significant differences in the frequency of bacteria that were natively coated with total IgG or with IgG4, nor did we find any differences in IgG or IgG4 staining when the bacteria were incubated with autologous serum regardless of whether we looked at all bacteria (Fig. 4A) or only those with low DNA (Fig. 4B). We observed a wide range of staining patterns in both IgG4-RD and HC samples (Fig. 4C), perhaps reflecting differences in the individual taxonomic composition. In summary, antibodies of IgG4 isotype themselves apparently do not directly contribute to shaping the microbial signature in patients with IgG4-RD.

## Discussion

Here, we have characterised the intestinal microbiota in a cohort of patients with IgG4-RD by conventional 16S rRNA gene amplicon sequencing, combined with single cell-based analysis of host immunoglobulin coating and surface sugar expression, in comparison to healthy individuals. We demonstrate that the microbiota of patients with IgG4-RD exhibits taxonomic alterations and decreased diversity. However, these features were not reliably sufficient for patient classification. In contrast, the IgG4-RD associated distinct microbiota phenotype, predominantly characterised by DNA^low^ bacteria, was identified as a robust feature for patient identification by machine learning.

Similar to previous studies,^11^ our IgG4-RD cohort was characterised by an increase in abundance of several taxa belonging to the phylum Bacillota (Firmicutes) and a decrease in several *Bacteroides* compared to HC. The IgG4-RD associated microbiota was equally rich as that of healthy donors, i.e. the number of different taxa is comparable; however, the relative distribution of these taxa was less “balanced” with increased shifts in abundance towards few taxa (decreased Shannon and Simpson diversity), typically seen in chronic diseases. Thus, the IgG4-RD microbiota was not defined by a significant loss of taxa as seen in other chronic inflammatory diseases, but rather by changes in relative abundances with Bacillota being the most prominent.

Phenotypically, the IgG4-RD microbiota contained clusters of cells that displayed a marked reduction in DNA staining and for some distinct clusters with high FSC signals an elevated coating with host immunoglobulins IgA1, IgA2, IgM and IgG isotype compared to that of healthy donors. While more detailed analyses and validation in larger cohorts will be necessary, this finding might indicate that IgG4-RD is associated with changes in the mucosal immune response, apparently irrespective of the IgG4-RD clinical phenotype. The decreased DNA staining could point towards a functional consequence of alterations in the intestinal environment due to disease. In other studies, the accumulation of e.g. pancreatic metabolites has been described for IgG4-RD, which may depress the overall metabolic function of the intestinal microbiota. Concomitantly, a decrease in metabolic activity has been associated with decreased DNA staining^34^^,^^35^ and is also found in Crohn's disease.^7^^,^^36^

The changes in gut microbiota we identified were sufficient to train machine-learning based models for the classification of IgG4-RD samples and healthy controls. However, the classification model based on 16S rRNA sequencing could not be validated with independently collected IgG4-RD samples. The limited performance in the validation step could be due to the different composition of IgG4-RD phenotypes in the training and validation cohort and the high interindividual diversity in microbiome composition.^37^ In fact, none of the selected, enriched taxa for IgG4-RD were present in every donor of the training cohort, which most likely prohibits the transferability of the signature to all individuals. As the age-distribution of the validation cohort differed from that of the test cohort, we cannot exclude that age-related changes in the microbiome prohibited validation. However, it may also indicate that it is not the gain or loss of particular taxa which govern the dysbiosis in IgG4-RD but rather changes in functionality which cannot be directly attributed to taxonomic changes, with the important limitation that we did not consider other microorganism, such as fungi or viruses. Correspondingly, the classification model using the phenotypic signature, which potentially reflects more functional changes, could be validated with the independent cohort. When combining the phenotypic signature with the 16S rRNA sequencing for classification, we did not see any improvement in the model. In fact, following the selection of features by Wilcoxon and RFE, only phenotypic features remained, indicating that 16 S rRNA sequencing does not contribute any additional information to the classification model (data not shown). Thus, despite the heterogeneity within our IgG4-RD cohort regarding the organs involved, therapy, antibiotics history and age, we could identify phenotypic features of the intestinal microbiota common to all patients with IgG4-RD. This is even more striking considering that only four out 28 patients in the training cohort and no patients in the validation cohort had an involvement of the gastrointestinal tract. Thus, our results support a prominent hypothesis in IgG4-RD, that the different organ manifestations of IgG4-RD are the result of a skewed systemic immune response, rather than just a shift in the local microenvironment of the affected organ.

Our data opens the speculation that the systemic effect on the immune system is the consequence of alterations in the intestinal microbiota. However, we cannot attribute such an effect to IgG4, the hallmark of IgG4-RD, as we did not find any relation between the microbiota and IgG4, neither in native staining nor after incubating microbiota with autologous patient serum. IgG4-RD is characterised by systemic type 2 and regulatory immune signature, including elevated IL-4, IL-5, and IL-13, together with increased IL-10 and TGFβ. This cytokine milieu is increasingly recognised as relevant to host–microbiota interactions, as these mediators modulate epithelial barrier function, antimicrobial interactions, and immune tolerance towards commensal organisms. In addition, innate immune activation, including eosinophils and macrophages responses driven by pattern-recognition receptor signalling, further links microbial sensing pathways to systemic immune activation.^38^^,^^39^ A recent study in IgG4-RD ophthalmic disease demonstrated significant gut microbial composition changes, including enrichment of opportunistic taxa and depletion of short-chain fatty acid-producing commensals such as *Faecalibacterium*, together with significant correlations between microbial taxa and circulating IgG4-RD-associated inflammatory markers, including a positive correlation between *Bifidobacterium* and IL-5.^40^ In this context, the distinct microbiome signatures identified in our study may represent downstream manifestations of systemic immune dysregulation, although this remains highly speculative and requires further validation. Moreover, population-level analyses suggest that systemic IgG4 levels are associated with gut microbial composition in humans, indicating bidirectional immune–microbial interactions, even in non-disease states.^41^

Our study has several limitations. First, the number of recruited patients was relatively low, and the study was performed at a single-centre. Second, at the time of analysis, the majority of patients with IgG4-RD were under active immunosuppressive or biologic treatment with rituximab and only 5 out of 28 patients (18%) were treatment-naïve. In combination with the small cohort size of patients with IgG4-RD and heterogeneous clinical manifestations, which limit statistical power, the effect of therapy on the microbiota may have confounded the presented results and reduce their generalisability. A further limitation is that IgG4-RD training and validation cohorts were not age-matched with each other or with their healthy controls. Age has been identified as a potential confounder in microbiome studies, particularly in childhood diseases, and as a major determinant of microbiome composition.^33^ However, the patient cohorts were balanced in terms of clinical and serologic features. Finally, the validation cohort was relatively small and the obtained results should be regarded as proof-of-concept rather than fully validated findings. Further independent validation will be required before clinical implications can be considered.

In summary, we present a comprehensive analysis of the intestinal microbiome in IgG4-RD that characterises not only the bacterial composition but also the microbiota phenotype. We have identified relevant differences in the intestinal microbiome of patients with IgG4-RD, and found more robust phenotypic alterations, which may support the identification of IgG4-RD independently of the disease phenotype. However, the relatively small number of patients within each subtype limits our ability to fully exclude subtype-specific effects. The altered microbiota phenotypes appear to be indicative of an altered immune response in the gut and adaptation of the bacteria to the disease-associated microenvironment. We did not find any indication that the elevated IgG4 response is directly linked to the microbiome. Our data suggest that microbiota fingerprinting combined with machine learning may have the potential for diagnosing IgG4-RD; however the current evidence is not sufficiently robust and validated to replace tissue biopsy, which remains the diagnostic gold standard. The robustness of the obtained features should be tested in larger, multicentre cohorts, including a greater number of treatment-naive patients and disease control groups with similar disease pathophysiology or clinical presentation. Studies that would combine the direct isolation of bacteria with a more detailed functional characterisation may help to reveal a mechanistic link between IgG4-RD and the intestinal microbiome in the future.

## Contributors

LB, AEB, HDC and TA conceived and designed the study. AEB and TA collected samples and performed the clinical characterisation of the patients. LB, AEB, TS, LL, RM, MFM and AA were responsible for the data acquisition (platforms) and analysed the samples. LB & GUK analysed and visualised the data. All authors interpreted the data. LB, AEB, HDC and TA wrote the first version of the manuscript and all authors critically revised and approved the final version. LB, HDC and TA had full data access and verified the underlying data. All authors had final responsibility for the decision to submit for publication. LB, AEB and TS, and HDC and TA contributed equally to this work.

## Data sharing statement

Source data for the figures are deposited with this paper. 16S rRNA gene sequencing data, as well as microbioata flow cytometry data, are available at Zenodo-Identifier 10.5281/zenodo.13684879.

## Declaration of interests

TA declared honoraria from Amgen, AstraZeneca, GSK and Zenas and study support from Johnson & Johnson. The others declared no potential conflict of interest.
