# Supplementary figures and images for: IgG4-related disease has a specific intestinal microbiota signature
Source: eBioMedicine. 2026 Jun 11;129:106326. doi: 10.1016/j.ebiom.2026.106326 (PMC13276518; doi:10.1016/j.ebiom.2026.106326)

Fig. S1

A

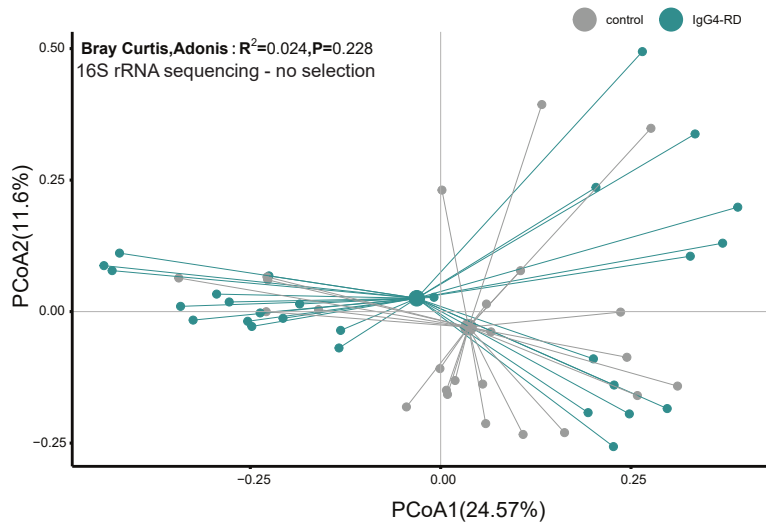

B

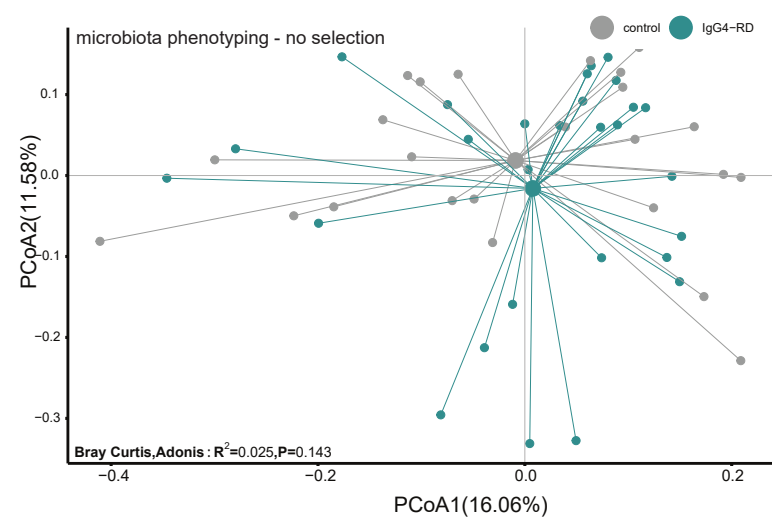

Fig. S2

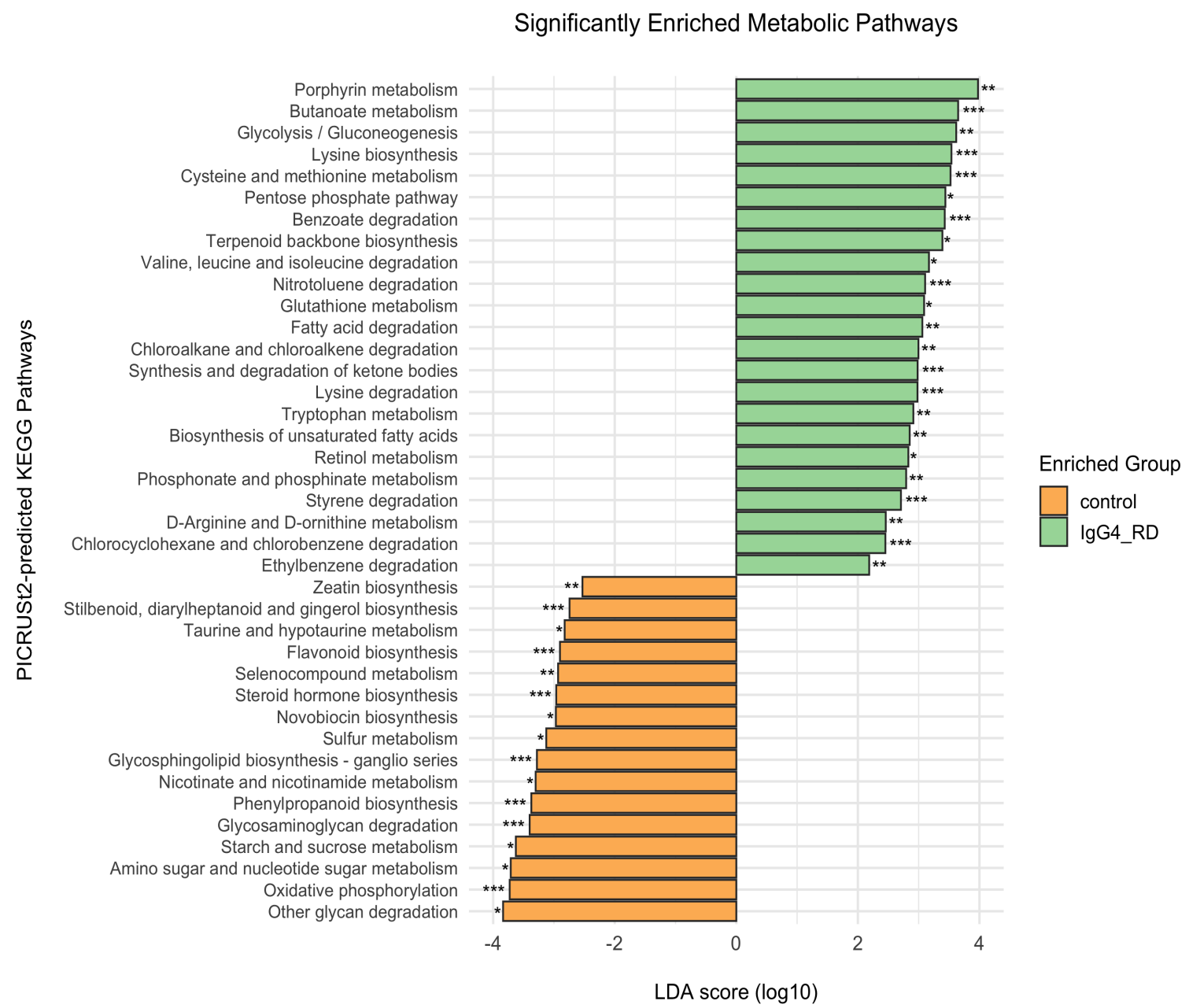

Fig. S3

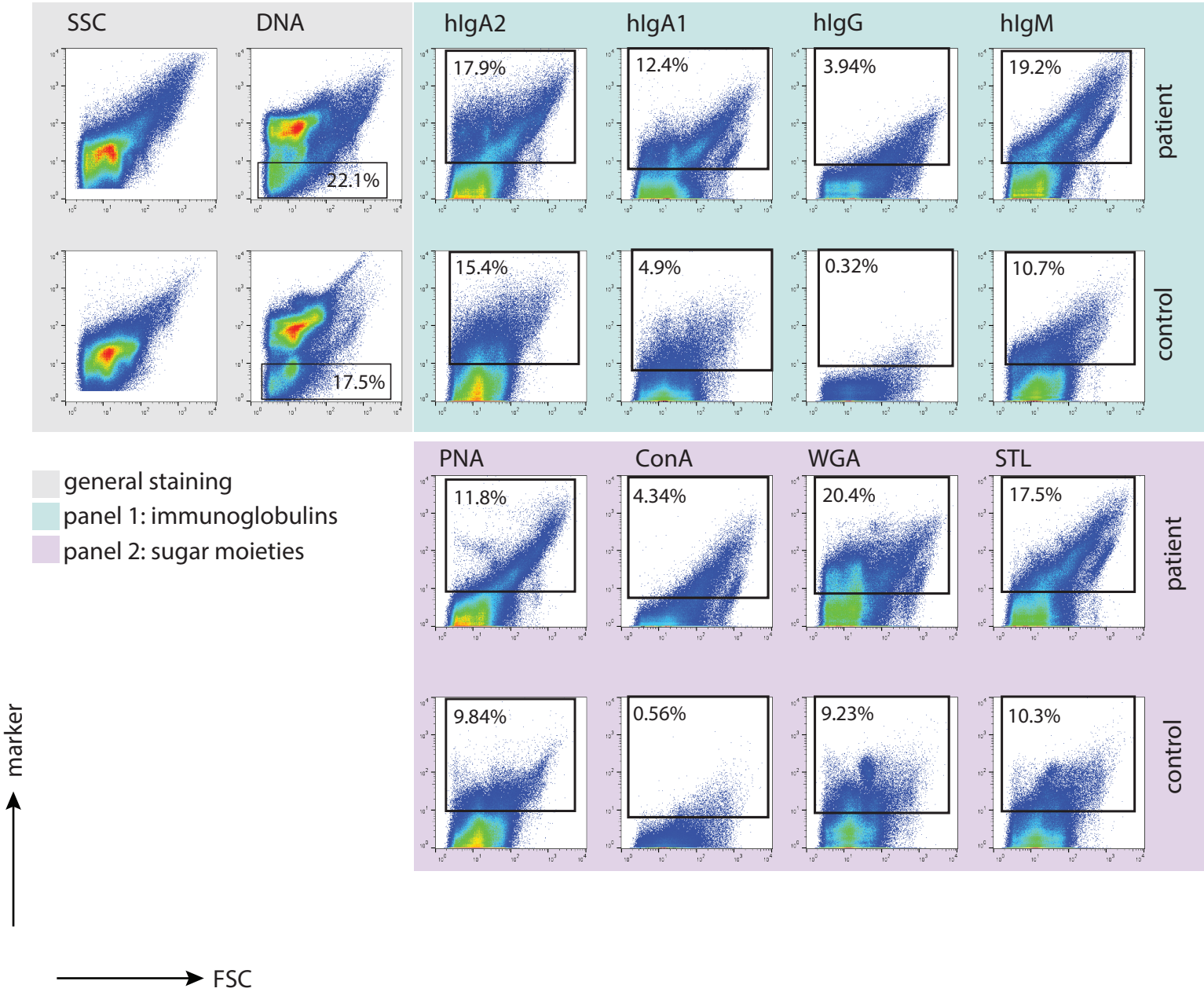

Fig. S4

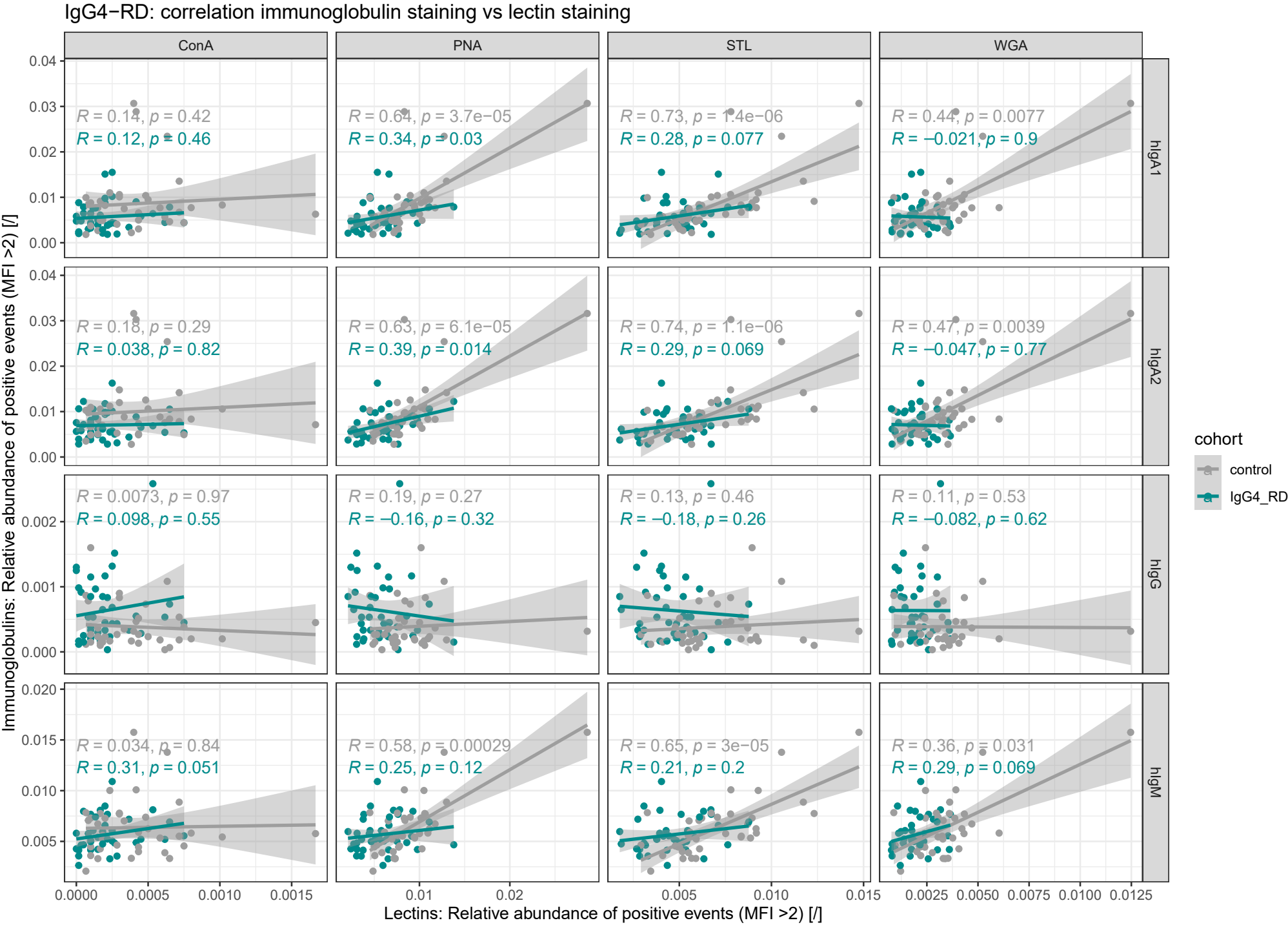

Fig. S5

A

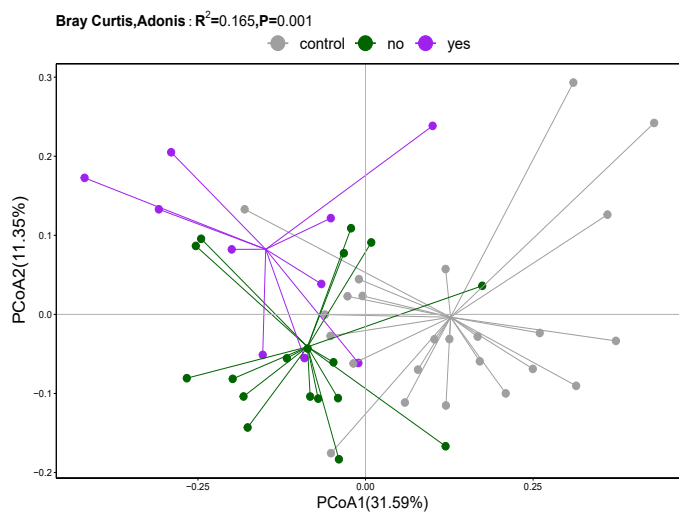

B

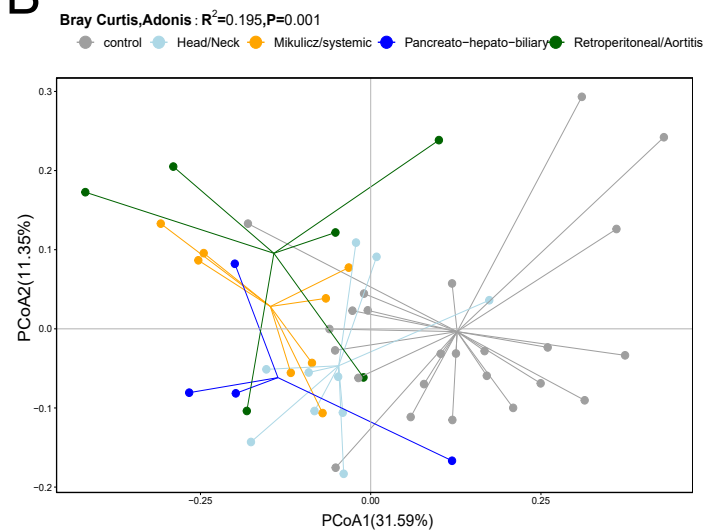

C

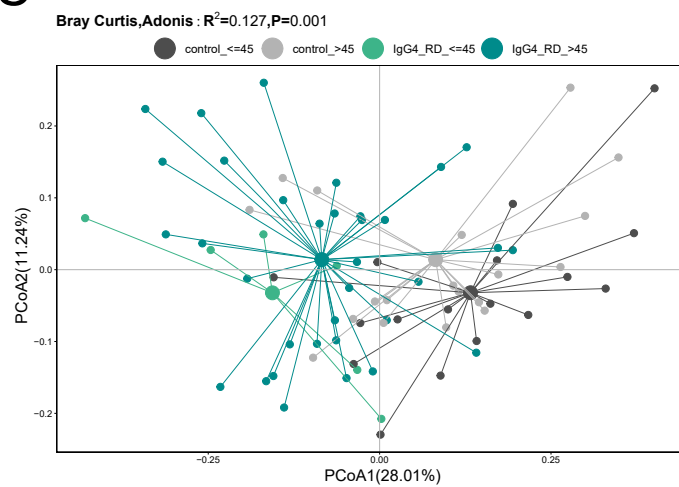

D

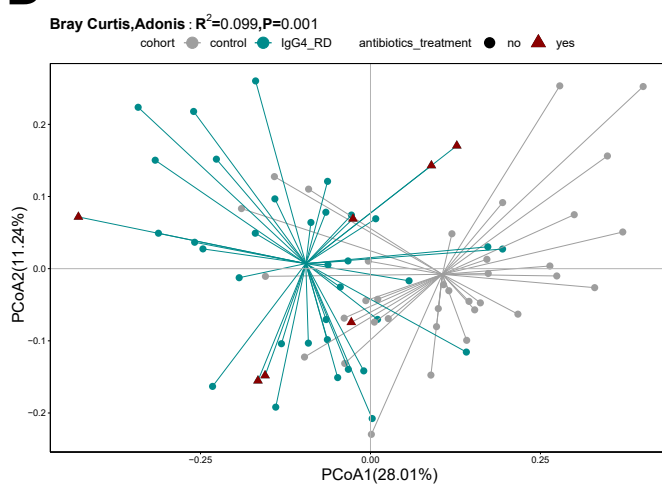

Supplement: Supplementary Figures [file mmc1.pdf]
