# Supplementary material for: IgG4-related disease has a specific intestinal microbiota signature
Source: eBioMedicine. 2026 Jun 11;129:106326. doi: 10.1016/j.ebiom.2026.106326 (PMC13276518; doi:10.1016/j.ebiom.2026.106326)
Supplement: Supplementary Table [file mmc2.pdf]

Table S1

A. Training cohort (IgG4 and HC)

	**IgG4-RD n=28**	**Healthy Controls n=24**	**p-value**
Age (median years, range)	55 (29-83.1)	47.4 (26.0-75.0)	0.002*
Male (n, %)	18 (64.3)	16 (66.7)	0.857^1
Female (n, %)	10 (35.7)	8 (33.3)	0.857^1

B. Validation cohort (IgG4 and HC)

	**IgG4-RD n=12**	**Healthy Controls n=12**	**p-value**
Age (median years, range)	70.3 (47-83)	50.3 (26-65)	0.002*
Male (n, %)	5 (41.7)	5 (41.7)	1^1
Female (n, %)	7 (58.3)	7 (58.3)	1^1

Supp. Table 1. Comparison of demographic of the training (A) and validation (B) cohorts for IgG4-RD patients and healthy controls (HCs) *Mann-Whitney-U Test, ^{1}Pearson Chi-Square.
